# Building Caregiver Resilience: Pitfalls and Potential for Interventions

**DOI:** 10.1093/geroni/igab046.202

**Published:** 2021-12-17

**Authors:** Jeongeun Lee, Natasha Peterson, Steven Zarit

## Abstract

Informal caregivers provide substantial practical and emotional support for individuals with chronic and acute conditions. Consequently, many experience caregiver burden and are at high-risk for psychological morbidity and associated breakdown in the provision of care for care recipients. Many psychosocial interventions have been designed to help caregivers. However, more work is needed to identify which, or what kind of, interventions are optimal for identifying suitable strategies and care management. The main objectives of this symposium are to (1) address psychosocial and demographic factors contributing to caregiver resilience, (2) understand the role of cognitive and behavioral factors that have implications for caregivers’ psychological well-being, and (3) specify different caregiving styles and adaptive outcomes. This symposium assembles a panel of experts and brings together empirical research on various challenges that need to be addressed and potential opportunities for creating effective psychosocial intervention targets for caregivers. The first session will discuss several psychosocial and demographic factors associated with resilience among caregivers. The second session will share how caregiving appraisals are closely related to positive and negative affect and whether the level and changes in caregivers’ activity participation moderate this linkage. The third session will identify caregiving styles and strategies utilizing k-modes machine learning analysis and share how caregivers adapt to care situations. The final session will present caregivers’ stress experiences related to dementia patients’ behavior and psychosocial symptoms in dementia during the day. The session will conclude with Dr. Zarit, who will integrate the four papers and offer insight on implications across studies.

